# Are anti-HIV IgAs good guys or bad guys?

**DOI:** 10.1186/s12977-014-0109-5

**Published:** 2014-12-14

**Authors:** Mingkui Zhou, Ruth M Ruprecht

**Affiliations:** Department of Virology & Immunology, Texas Biomedical Research Institute, 7620 NW Loop 410, San Antonio, TX 78227 USA; Southwest National Primate Research Center, 7620 NW Loop 410, San Antonio, TX 78227 USA

**Keywords:** IgA, Dimeric IgA1 (dIgA1), dIgA2, Secretory IgA (SIgA), HIV/SHIV, Mucosal transmission, Passive immunization, Non-human primate models, RV144 trial, AIDS vaccine development

## Abstract

An estimated 90% of all HIV transmissions occur mucosally. Immunoglobulin A (IgA) molecules are important components of mucosal fluids. In a vaccine efficacy study, in which virosomes displaying HIV gp41 antigens protected most rhesus monkeys (RMs) against simian-human immunodeficiency virus (SHIV), protection correlated with vaginal IgA capable of blocking HIV transcytosis in vitro. Furthermore, vaginal IgG exhibiting virus neutralization and/or antibody-dependent cellular cytotoxicity (ADCC) correlated with prevention of systemic infection. In contrast, plasma IgG had neither neutralizing nor ADCC activity. More recently, a passive mucosal immunization study provided the first direct proof that dimeric IgAs (dIgAs) can prevent SHIV acquisition in RMs challenged mucosally. This study compared dimeric IgA1 (dIgA1), dIgA2, or IgG1 versions of a human neutralizing monoclonal antibody (nmAb) targeting a conserved HIV Env epitope. While the nmAb neutralization profiles were identical in vitro, dIgA1 was significantly more protective in vivo than dIgA2. Protection was linked to a new mechanism: virion capture. Protection also correlated with inhibition of transcytosis of cell-free virus in vitro. While both of these primate model studies demonstrated protective effects of mucosal IgAs, the RV144 clinical trial identified plasma IgA responses to HIV Env as risk factors for increased HIV acquisition. In a secondary analysis of RV144, plasma IgA decreased the in vitro ADCC activity of vaccine-induced, Env-specific IgG with the same epitope specificity. Here we review the current literature regarding the potential of IgA – systemic as well as mucosal – in modulating virus acquisition and address the question whether anti-HIV IgA responses could help or harm the host.

## Introduction

Mucosal secretions represent the first line of defense to protect a host against invasion of viral pathogens, including HIV. Dimeric or polymeric IgA molecules are important components of mucosal fluids; IgA is present in vaginal and rectal secretions, in saliva, gastric fluid, tears, sweat and in colostrum as well as mature milk. Monomeric IgA is also one of the major immunoglobulin classes present in serum, second only to IgG. In humans, the production of IgA per day is greater than that of the other classes of immunoglobulins combined [1]. Despite this, the ability of IgA – both systemic and mucosal – to modulate the risks of HIV infection remains relatively understudied.

## Review

### Structure and subclasses and of IgA

**Figure 1:**
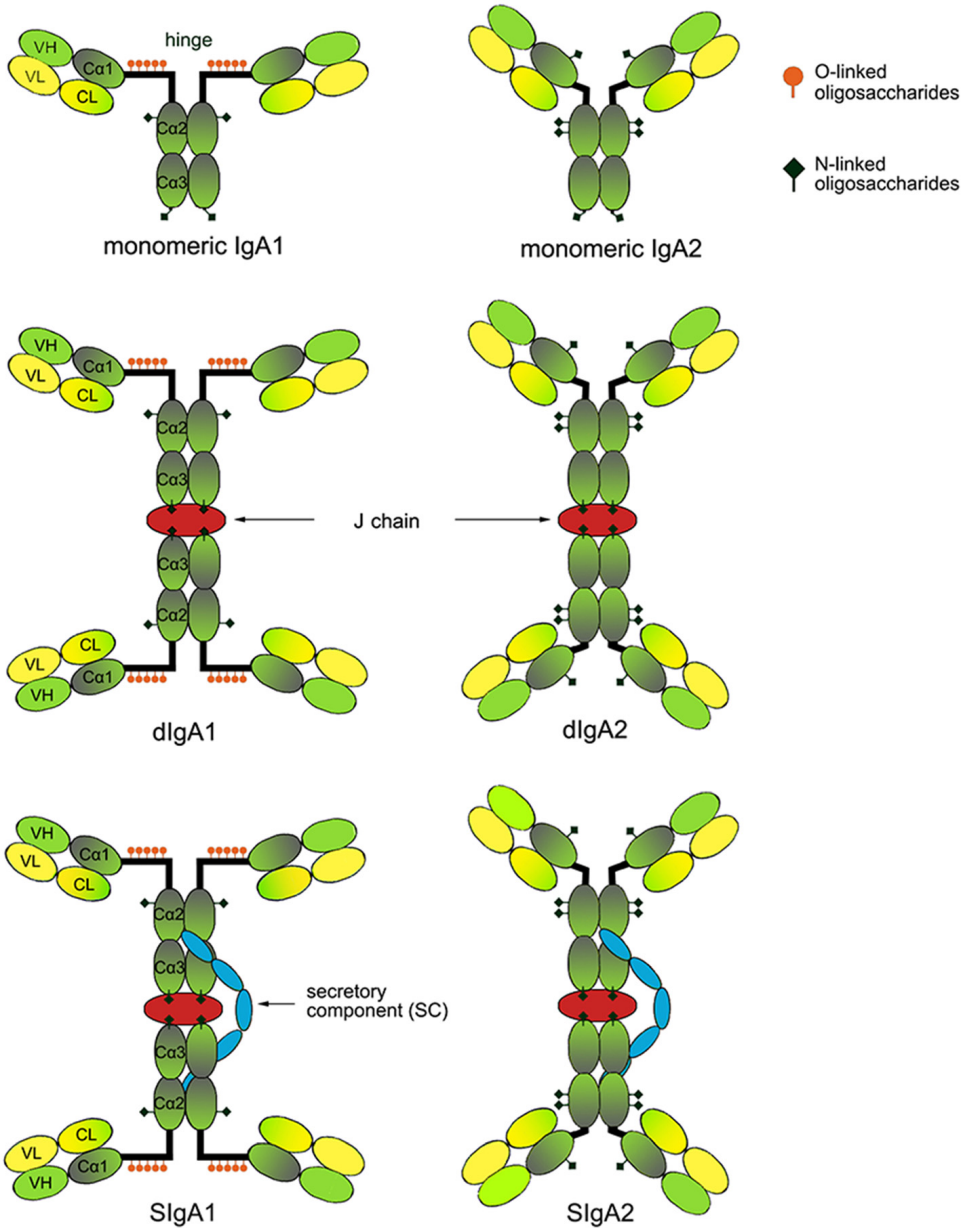
**Human IgA1 and IgA2 molecules as monomers, dimers (dIgA1 and dIgA2, respectively) and as secretory forms (SIgA1 and SIgA2, respectively).** Green, heavy chain; yellow, light chain; red, J chain; blue, secretory component (SC); orange, O-linked oligosaccharides in the IgA1 hinge region. N-linked oligosaccharides are shown at the approximate locations in both IgA1 and IgA2 molecules.

The structure of IgA is similar to that of other immunoglobulins. Monomeric IgA consists of two heavy chains and two light chains, which are stabilized by non-covalent interactions. Each heavy chain is composed of four subdomains: the variable region of the heavy chain (VH), and the constant regions α1 (Cα1), Cα2 and Cα3, whereas the light chains have two subdomains, the variable region of the light chain (VL) and the constant region (CL) (Figure 1).

Human IgA has two closely related subclasses, termed IgA1 and IgA2; the major difference between these two lies in the hinge region (Table 1). In IgA1 molecules, the hinge region contains 19 amino acids (aa) [2] and a number of O-linked oligosaccharides [3,4], whereas the hinge region of IgA2 molecules is only 6 aa long [2] and lacks glycosylation [5]. As a consequence of their open hinge region, IgA1 molecules have a T-like shape, in which the distance between Fab fragments measures approximately 16 nm [6-8]. In contrast, IgA2 is Y-shaped, and the distance between Fab regions is only 10 nm due to the shorter and stiffer hinge region [7-9]. The structural differences between IgA1 and IgA2 molecules are likely associated with differential biological activities.

**Table 1:** **IgA Cα gene in different mammalian species**

**Species (number of Cα genes)**	**Cα Gene**	**Hinge length region (aa)**	**References**
Human (2)	**Cα1**	19	[2,10]
	**Cα2**	6	
Gorilla (2)	**Cα1**	19	[2,10-12]
	**Cα2**	6	
Chimpanzee (2)	**Cα1**	19	
	**Cα2**	6	
Orangutan (1)	**Cα**	17	
Gibbon (2)	**Cα1**	9	
	**Cα2**	6	
Rhesus macaque (1)	**Cα**	7	[2,10-12]
Baboon (1)	**Cα**	7	
Rabbit (13)	**Cα1**	10	[2,13-17]
	**Cα2**	23	
	**Cα3**	23	
	**Cα4**	23	
	**Cα5**	23	
	**Cα6**	23	
	**Cα7**	10	
	**Cα8**	Not expressed	
	**Cα9**	10	
	**Cα10**	15	
	**Cα11**	10	
	**Cα12**	8	
	**Cα13**	22	
Mouse (1)	**Cα**	12	
Pig (1)	**Cα**	9	
Bovine (1)	**Cα**	9	
Dog (1)	**Cα**	11	

In human serum, approximately 90% of IgA consists of IgA1 and 10% of IgA2 [8]. In contrast, the ratio of IgA1 and IgA2 varies in different mucosal fluids, with IgA1 percentages in male genital secretions and nasal fluids reaching 80-90% and 60% in saliva [18]. Female genital secretions and rectal fluids contain approximately 60% IgA2. Human colostrum was reported to have even higher ratios of IgA2 compared to IgA1; the concentrations of both components decreased during the time of lactation to significantly lower levels in mature milk [19].

IgA in serum is mainly monomeric with dimeric or polymeric forms ranging from <1% to 20% [2]. Polymeric forms of serum IgA include trimers and tetramers.

In mucosal fluids, the major IgA form is secretory IgA (SIgA). It is generated from dimeric (dIgA) secreted locally from mature mucosal plasma cells; dIgA consists of two IgA monomers linked covalently via their Fc portions to the joining (J) chain. The secretory component (SC) is added during the passage of dIgA across the epithelial layer (see below). The open hinge region in SIgA1 makes this molecule more susceptible than SIgA2 to proteolytic cleavage by proteases derived from bacterial pathogens, such as *Haemophilus influenzae* and *Neisseria meningitidis* [20-22]. It is currently not known whether SIgA1 and SIgA2 exhibit differential susceptibility to proteolytic cleavage by normal microbial flora in the various mucosal fluids.

### The generation of SIgA

**Figure 2:**
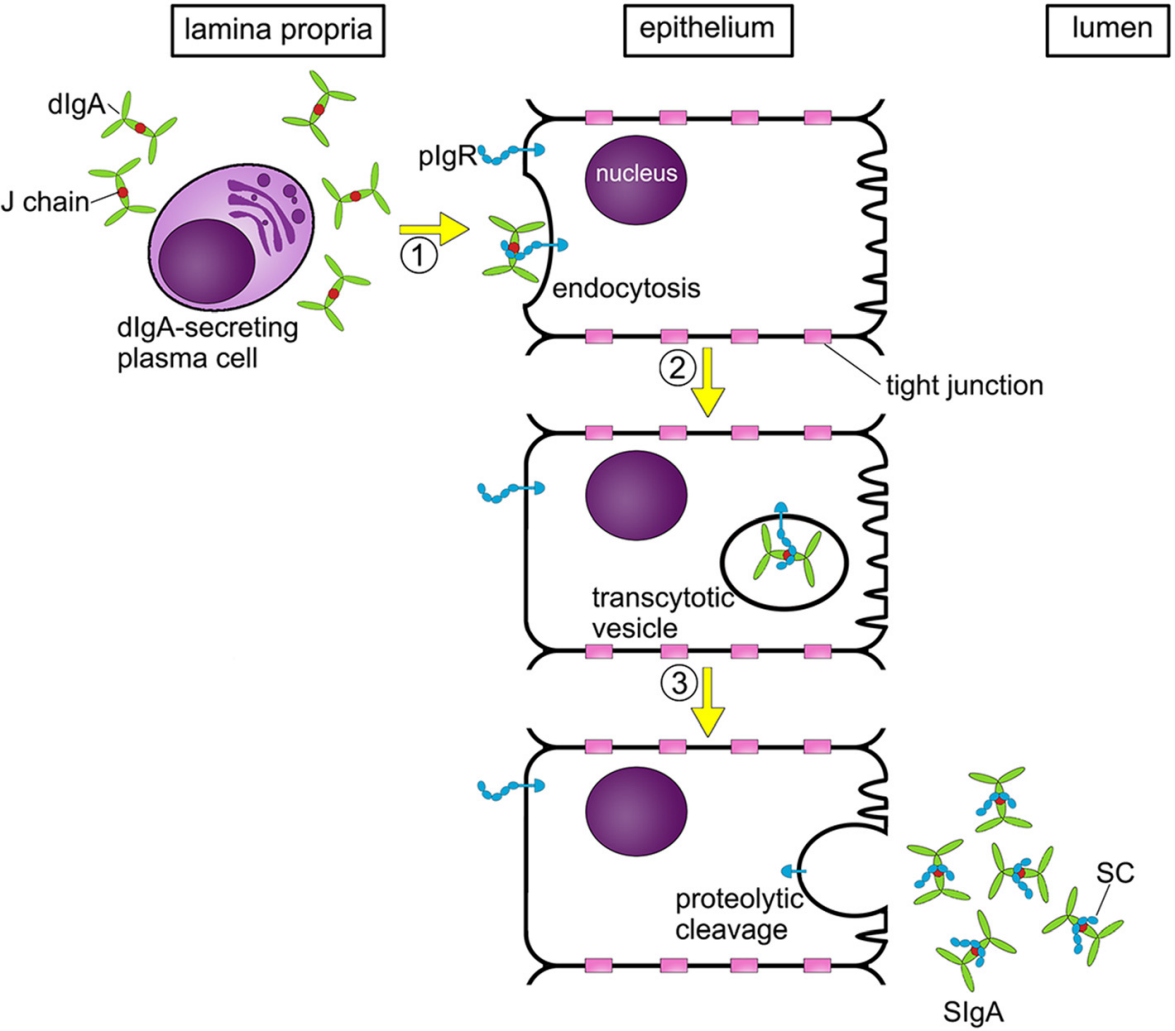
**Formation of SIgA.** Dimeric IgA (dIgA) is produced by mature plasma cells in the lamina propria; these cells also produce J chains. Step 1, dIgA interacts with the polymeric immunoglobulin receptor (pIgR; shown in blue) on the basolateral surface of epithelial cells. Step 2: export of dIgA across the epithelial cells is mediated by pIgR. Step 3: pIgR undergoes proteolytic cleavage at the luminal side, which results in the generation of secretory component (SC) that is retained by dIgA molecules, giving rise to secretory IgA (SIgA).

In contrast to serum IgA, which is derived from plasma cells in the bone marrow, SIgA is generated locally by plasma cells located in the lamina propria below the epithelium; these cells secrete dIgA, including J chains. After release, the dIgA molecules bind to the polymeric immunoglobulin receptor (pIgR) [23,24], a transmembrane glycoprotein of the Ig superfamily with five extracellular domains expressed on the basolateral surfaces of mucosal epithelial cells (step 1, Figure 2). Following binding to pIgR, the dIgA-pIgR complex is endocytosed and transported across the epithelial cell in a vesicle (step 2, Figure 2). The J chain is crucial for the formation of the pIgR-dIgA complex and offers a binding site for the pIgR [25]. On the apical side, the complex is released into the lumen, a process during which proteases cleave off SC from the pIgR (step 3, Figure 2). The final product, SIgA, is released into the lumen either as dimer or higher-order multimers and likely interacts with mucus. Such interactions differ from those of IgG, which is also present in mucosal secretions [26]. It is also possible that SIgA1 and SIgA2 bind differentially to mucus, given their differences in structure and glycosylation patterns. Interestingly, free pIgR can also transcytose to the apical surface and undergo proteolytic cleavage, which results in the release of free SC into mucosal secretions [27-29].

### IgA in different species

IgA molecules have been identified in many mammalian species [30]. Most only encode a single Cα gene, thus giving rise to single IgA subclass. The number of Cα genes in different mammalian species is summarized in Table 1. Humans and some of the great apes encode IgA1 as well as IgA2 [31], whereas rhesus macaques and many other species only encode one subclass [11]. Of note, the species most frequently used to generate and analyze antibody responses, mice and rabbits, encode either one [32] or 13 Cα genes [13], respectively, thus not reflecting the human system. Consequently, the only potential animal model to study differential IgA subclass responses may be chimpanzees.

## Methods to isolate various forms of human IgA

When evaluating existing literature regarding human IgA responses, technical issues need to be considered. Most publications do not distinguish between IgA1 and IgA2, and many also do not differentiate between monomeric, dimeric, or polymeric forms [33-39]. Furthermore, some studies only report on serum IgA responses, whereas others exclusively focus on IgA in mucosal fluids. Much needs to be learned about the dynamics and specificities of IgA responses in the systemic circulation and their relationship to IgA responses in mucosal compartments. Current IgA isolation methods are given in Table 2. It will be important to reassess the distribution of IgA1 and IgA2 in different human mucosal fluids with reagents and methods with equal sensitivity, specificity and recovery for both IgA subclasses.

**Table 2:** **Human IgA isolation methods**

**Reagent for IgA isolation**	**IgA site recognized**	**IgA form recognized**	**References**
Jacalin resin	α-D-galactose (O-linked) in the IgA1 hinge	Preferential binding to all IgA1 forms; Weak binding to IgA2	[33,34,37]
Staphylococcal superantigen-like protein 7	Fc	Specific for all IgA1 and IgA2 forms	[36,39]
Streptococcal IgA-binding peptide	Fc	Specific for all IgA1 and IgA2 forms	[35,38]
**mAbs**			
Anti-J chain mAb	J chain	Specific for dimeric and secretory forms of IgA1 and IgA2	Commercially available
Anti-SC mAb	SC	Specific for secretory forms of IgA1 and IgA2	
Specific anti-IgA1 mAb	Constant region	Specific for IgA1	
Specific anti-IgA2 mAb	Constant region	Specific for IgA2	

Methods are available to isolate SIgA with reagents specific for SC. The fact that the open hinge region in IgA1 makes this molecule more susceptible to proteolytic cleavage by proteases of pathogenic bacteria could also be exploited for the selective isolation of SIgA2. To differentiate between IgA1 and IgA2 versions of the various forms of IgA, monoclonal antibodies (mAbs) are commercially available that show a high degree of specificity for either human IgA subclass.

In summary, it is important to review the IgA isolation methods when judging existing literature on IgA. Much analytical work remains to be carried out to determine the roles of various human IgA forms – monomeric versus dimeric or multimeric, systemic versus mucosal, as well as IgA1 versus IgA2 – in modulating HIV transmission.

### Unanswered question regarding secretory IgA (SIgA)

A number of questions regarding the biology of SIgA responses have not yet been fully addressed, especially in humans and non-human primates. Important aspects of SIgA biology have been studied in mouse models (reviewed in [40]). According to this review, key questions that remain to be addressed include i) the longevity of mucosal plasma cells, ii) the need for T-cell involvement, iii) the ability of B cells to undergo class switch recombination locally in the lamina propria and iv), innate versus adaptive IgA responses. It is especially important to address such questions in non-human primate models.

Studies in mice lacking T-cell immunity have indicated that some mucosal IgA responses against intestinal commensal bacteria were retained [41], implying their T-cell independence. Antigen sampling at specialized mucosal sites, the trafficking of B cells, and the generation of SIgA at different mucosal sites has been reviewed by Neutra and Kozlowski, especially with regards to induction of adaptive mucosal immune responses by vaccination [42].

### IgA interactions with Fc receptors

Like IgG, IgA interacts with cell surface-expressed Fc receptors, the best known one being the Fc receptor αRI (FcαRI) [43], also known as CD89. FcαRI is encoded by a single gene [44] and is expressed on monocytes/macrophages, dendritic cells, neutrophils, eosinophils, and Kupffer cells of the liver [45,46]. Through interactions with FcαRI, IgA can activate (ADCC) [47] and complement [48]. In addition, the FcαRI-IgA interactions can also result in cytokine secretion by the receptor-bearing cells and modulate phagocytosis. IgA can also interact through its Fc moiety with Fcα/μR, known as CD351 [49,50], which is expressed on mature B cells, macrophages, Paneth cells [51] and on follicular dendritic cells [52]. The consequences of the Fcα/μR-IgA interaction remain to be elucidated. Of note, the standard ADCC assays using NK cells as effector cells are not expected to be positive for IgA-mediated ADCC since NK cells express neither CD89 nor CD351.

## Protective Mechanisms of IgA in viral infections

### Direct neutralization, immune exclusion and inhibition of transcytosis

**Figure 3:**
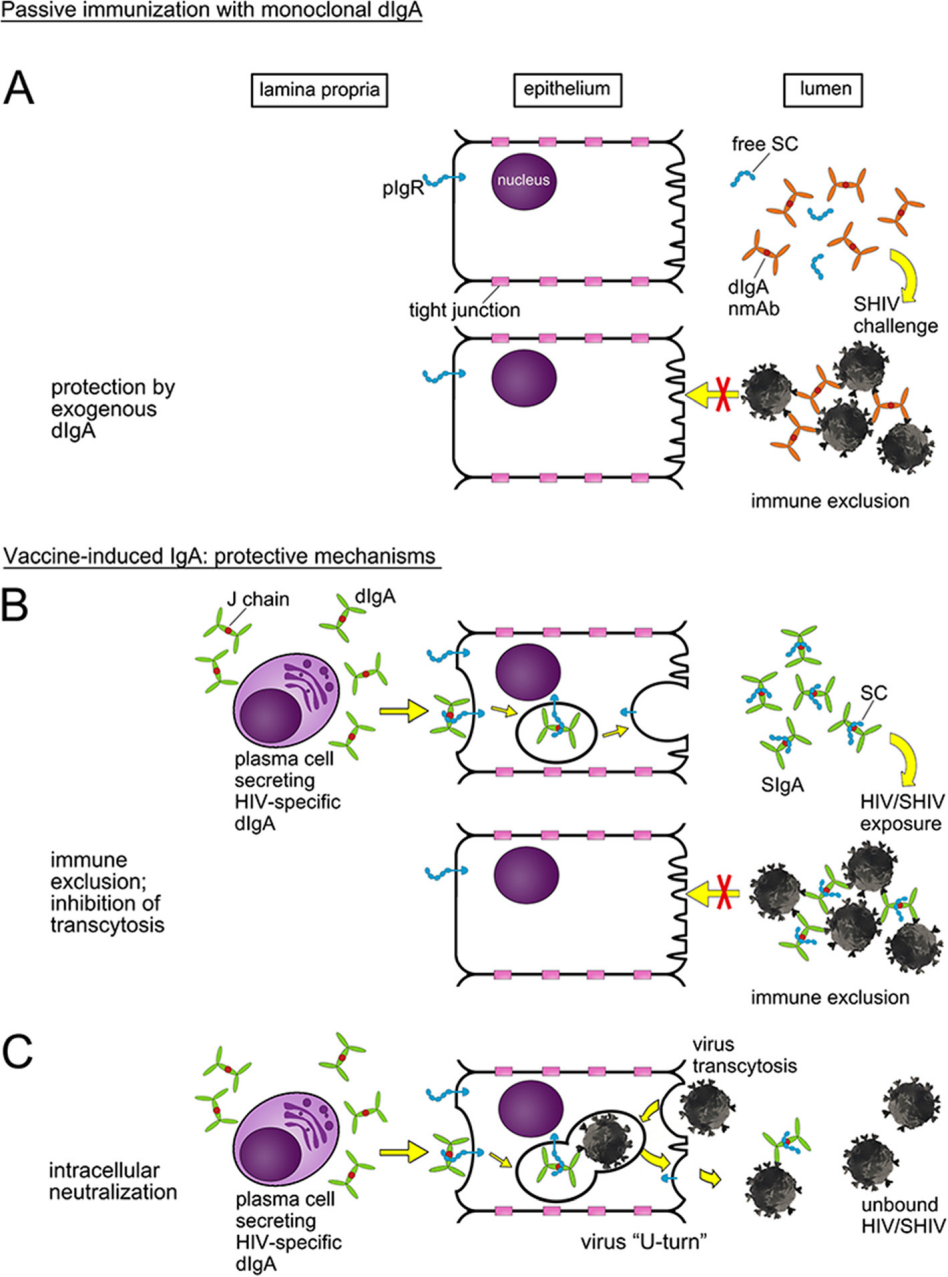
**IgA-mediated protection against HIV/SHIV at mucosal surfaces. A**. Passive immunization with a neutralizing monoclonal dIgA. After topical administration of the neutralizing mAb in the mucosal lumen of macaques, dIgAs bind to SHIV and prevent virions from crossing the epithelial barrier by forming large SHIV-dIgA complexes. Reports from the literature describe the presence of free SC in various mucosal fluids [27-29]. **B** and **C**. Protection by locally produced IgA in HEPS individuals or induced by vaccination. **B**. Immune exclusion; inhibition of transcytosis. HIV/SHIV-specific dIgA is produced by mature plasma cells in the lamina propria and interacts with pIgR (in blue) on the basolateral surface of epithelial cells; export of dIgA across the epithelial cells is mediated by pIgR. The latter undergoes proteolytic cleavage at the luminal side, which results in the generation of SC that is retained by dIgA molecules, releasing SIgA into the lumen. SIgA binds to SHIV and prevents viral invasion of epithelial cells by forming large SHIV-dIgA complexes. **C**. Intracellular neutralization. After the dIgA-pIgR complex is formed intracellularly, HIV/SHIV particles that have invaded the epithelial cells are bound and then excreted as virion-dIgA-pIgR complex. This returns the SIgA-virion complex into the mucosal lumen.

In mucosal fluids, SIgA provides the first line of defense against invading viruses, as IgA can either directly neutralize virions, mediate their adherence to mucin glycoproteins, or aggregate them into large IgA-virion complexes; interactions with mucus serve to retain virions in the lumen, a phenomenon termed “immune exclusion”. A recent passive immunization study [53] provided proof-of-principle that dIgAs given directly into the rectal lumen as neutralizing monoclonal antibodies (nmAbs) can prevent SHIV acquisition (Figure 3A). Although the exogenously administered dIgAs did not contain SC, they may have associated with free SC, which is present in mucosal secretions [27-29].

Vaccine-induced mucosal IgA responses can also block HIV or SHIV infection after mucosal exposure (Figure 3B). Plasma cells in the lamina propria secreting HIV-specific dIgA play an important role in this protective mechanism; after secretion from lamina propria plasma cells, anti-HIV dIgA binds to pIgR followed by uptake into a transcytotic vesicle. HIV-specific SIgA is released on the luminal side after proteolytic cleavage of pIgR and retention of the secretory component to yield SIgA. The data suggest that SIgA can either directly neutralize incoming HIV or crosslink it in large virion-SIgA complexes [54-59], resulting in immune exclusion (Figure 3B).

### Intracellular neutralization

This mechanism was first described by Burns et al. [60] using a rotavirus murine model. Rotavirus can cause severe diarrhea in young children and animals. The authors discovered that two oligomeric IgA mAbs directed against VP6, a major inner viral capsid protein, could prevent infection after oral challenge of mice. The dIgA mAbs were inactive when administered directly into the lumen of the intestinal tract. The authors postulated that the non-neutralizing IgA mAbs encountered nascent viral proteins while being transported by pIgR across epithelial cells, known to support rotavirus replication. While these mAbs were non-neutralizing in standard assays, they were able to bind to VP6 in the endocytic vesicle during transepithelial transport and prevent virus assembly [60]. This process is termed intracellular neutralization.

### Mucosal IgA as mediator of immune excretion

As described above, dIgA binds to pIgR through the Cα3 region in the presence of the J chain. This interaction leaves the antigen-combining sites of the dIgA free. Consequently, the question arises whether dIgA carrying an antigenic cargo would still bind to pIgR and cross the epithelial cell layer from the apical to the luminal side. This question was addressed in vivo in a transgenic mouse system by Robinson et al. [61]. After intragastric immunization, the mice mounted strong mucosal antigen-specific IgA responses. After intravenous administration of the antigen, the latter was detected within epithelial cells of the small intestinal crypts and also within epithelial cells in more distal regions of the villi. Control mice immunized with an irrelevant antigen showed no such localization of the antigen. Antigen uptake by epithelial cells occurred only from the basolateral side in the presence of IgA-antigen complexes in the lamina propria [61]. These data imply that IgA-antigen complexes follow the same pIgR-mediated trans-epithelial movement as free dIgA.

**Figure 4:**
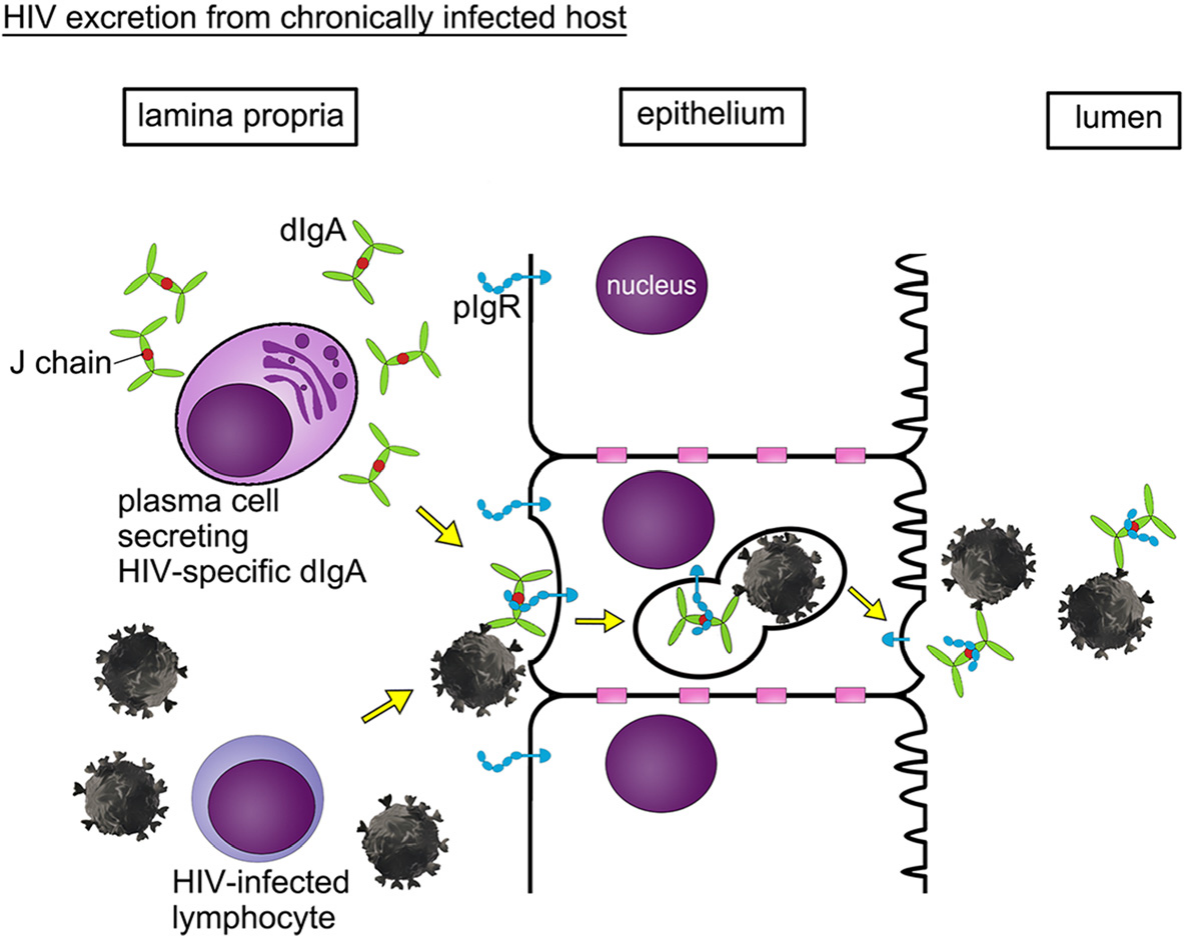
**Excretion of HIV from chronically infected hosts.** HIV-specific dIgA (shown in green) captures virions in the lamina propria, forming immune complexes that shuttle across the epithelial cell after binding to pIgR (shown in blue). The latter undergoes proteolytic cleavage at the apical site and releases an HIV-SIgA immune complex into the mucosal lumen.

The question then arose whether HIV particles complexed with dimeric or polymeric IgA could also be excreted by a pIgR-mediated mechanism (Figure 4). To test this notion, Wright et al. [62] used cell-culture systems consisting of either polarized epithelial cells stably expressing pIgR on the basolateral surface or human epithelial cell lines naturally expressing pIgR. A number of oligomeric IgA mAbs targeting either HIV gp41 or gp120 were tested; controls included the same mAbs in an IgG form or irrelevant anti-measles virus oligomeric IgA and IgG. These investigators placed mAb-HIV immune complexes into the bottom chamber in transwell plates and tested release of HIV particles into the upper chamber culture fluid overlaying the apical side of a tight epithelial layer. Transport of HIV particles was indeed observed and correlated directly with the ability of the IgA mAbs to bind to virions as well as to the pIgR. Excretion of HIV particles on the apical side required HIV-specific oligomeric IgAs and occurred at IgA concentrations that were in the range of those found in human mucosal fluids. Confocal microscopy showed co-localization of HIV antigens and anti-HIV oligomeric IgA within the polarized epithelial cells; control cells lacking pIgR expression did not facilitate transepithelial transport of HIV. It is currently not known whether the excretory function of dIgA contributes to the clearance of viral pathogens from inside the epithelial barrier and whether such a mechanism plays a role in the early stages of HIV invasion after mucosal exposure.

## Anti-HIV IgA responses linked to protection

### IgA responses in HIV-exposed, persistently seronegative persons (HEPS)

A number of studies examined mucosal antibody responses in HEPS, including mucosal IgAs [55,63-67]. Interestingly, studies that have linked HIV-specific IgA responses with resistance in sex workers and in persistently uninfected sexual partners of HIV-positive individuals have used jacalin-based methods to isolate mucosal IgAs. As mentioned previously, jacalin resins preferentially bind human IgA1 antibodies. In some instances, such IgA isolated by jacalin was also found in HEPS sera. Epitope mapping revealed that mucosal IgAs targeted relatively conserved epitopes in the membrane proximal external region (MPER) of HIV gp41 [68,69]. Cross-clade neutralization of such mucosal IgAs from HEPS has been described [63]. Of note, HIV-specific mucosal antibody responses were reported to be absent or detectable in only a low fraction of HEPS in several cohorts [70-73]. Consequently, a potential association of HIV-specific IgAs with remaining HIV negative is unclear. The discrepancy of findings regarding mucosal IgA in HEPS may be due to technical issues, including the addition of protease inhibitors to mucosal fluids, the use of jacalin-based IgA isolation methods, and assay sensitivity.

### Vaccine-induced mucosal IgA responses linked to protection of macaques against SHIV challenges

Virosomes displaying either an HIV gp41-derived peptide, P1, or a truncated recombinant gp41 were administered to Chinese-origin rhesus macaques (RMs) by intramuscular (i.m.) priming followed by either i.m or intranasal (i.n.) boosting. Only RMs vaccinated by the i.m./i.n. routes developed systemic as well as mucosal antibody responses. Four weeks after the last boost, the vaccinees were given a total of 13 low-dose intravaginal challenge with the heterologous tier 2 SHIV_SF162P3_. All macaques given empty virosomes became viremic and seroconverted. In contrast, four out of the five vaccinees given i.m. priming followed by i.n. boosting remained aviremic; a fifth animal had only transient low-level viral RNA blips. None of these five vaccinees seroconverted, indicating protection from persistent systemic infection. All of the protected RMs had developed gp41-specific IgAs in vaginal secretions that blocked transcytosis of HIV clade B and C strains in vitro. When the mucosal secretion of the vaccine-protected RMs were depleted either of IgA or IgG, the transcytosis inhibition was retained only when mucosal IgGs were depleted but not vice-versa, implying that vaccine-induced IgA was responsible for blocking HIV transcytosis in vitro [74].

The protected RMs also had developed vaginal IgG responses with neutralizing and/or ADCC activities. Interestingly, plasma IgGs did not neutralize the challenge virus. The authors concluded that vaccine-induced vaginal IgA responses together with IgG responses were linked to robust protection against vaginal SHIV challenges [74].

Inhibition of transcytosis by a primary HIV clade C strain implied that the combined virosome immunogens displaying either peptide P1 or recombinant, truncated gp41 shared epitope determinants that were recognized cross-clade. This is all the more interesting given that the primary amino acid sequences showed no linear epitope identity. In this context, it is noteworthy that Devito et al. [63] had described earlier HIV-blocking IgAs specific for the HIV gp41 MPER region in HEPS, and Tudor et al. [75] had detected P1-specific IgA responses in HEPS as well.

### Prevention of mucosal R5 SHIV transmission by neutralizing human monoclonal IgA1 and IgA2 in rhesus macaques

Direct proof that mucosal IgAs can prevent primate immunodeficiency virus transmission was provided by passive immunization with different isotypes of the human nmAb HGN194 that targets the conserved crown of the V3 loop in the HIV envelope glycoprotein [53]. dIgA1, dIgA2, and IgG1 versions of HGN194 were applied intrarectally (i.r.) to RMs 30 minutes before i.r. clade C SHIV challenge. A control pharmacokinetic study had demonstrated that the nmAb concentrations in the rectal fluids over time were similar for all three HGN194 isotypes, an important control study given that dIgA forms were used intrarectally. Unexpectedly, dIgA1 provided the best protection against i.r. SHIV challenge, despite the fact that all three nmAbs had similar neutralizing activity in vitro. Among RMs passively immunized with dIgA1, 83% were protected compared to only 17% of those given dIgA2 (*P = 0.045*). Better protection correlated significantly with virion capture; dIgA1 reproducibly captured twice as many virions compared to dIgA2 in vitro. Only dIgA1 blocked transcytosis of cell-free virus across an epithelial layer in vitro [53].

These data not only showed a direct correlation between dIgAs and prevention of mucosal SHIV transmission but also revealed a significant difference between the human dIgA1 and dIgA2 versions of the same nmAb that had not changed epitope specificity in the different backbones. These data and the results of Bomsel et al. [74] imply that AIDS vaccine strategies should focus on inducing mucosal IgA responses as a first line of defense against HIV, a virus predominately transmitted mucosally.

### Can IgA in breastmilk protect infants from milk-borne HIV transmission?

In areas of the world where infant mortality is high due to lack of resources and access to safe, affordable formula, exclusive breastfeeding for the first six months is recommended by WHO [76]. Studies have shown that exclusive breastfeeding is associated with a surprisingly low postnatal HIV transmission rate [77].

To test whether IgA in breastmilk of HIV-positive mothers was associated with protection of breastfeeding infants, a nested case–control study was performed in Zambia [78,79]. Total HIV Env-specific IgA levels were measured in milk samples collected from 26 HIV-infected mothers who transmitted the virus to their infants (transmitters) and from 64 mothers whose infants remained negative (non-transmitters). Overall, HIV Env-specific IgA in breastmilk was not linked to protection. HIV Env-specific IgA was detected more often in breastmilk of transmitting mothers compared to non-transmitting mothers. Unfortunately, the interpretation of the data was complicated by the fact that: “most infected infants in our study had detectable infection by 2 months of age, making it difficult to distinguish postpartum- and intrapartum-acquired infections” [78]; of the 26 infected infants, only four fulfilled the criteria of definite milk-borne mother-to-child transmission according to the authors. As a consequence, this study did not have sufficient statistical power to provide definite answers regarding a potentially protective role of IgA in milk for infants born to HIV-positive mothers and exclusively breastfed.

## Are Anti-HIV IgA responses harmful?

### IgA-dependent enhancement of HIV infection in vitro

Two separate studies reported enhancement of HIV replication in vitro by unfractionated, serum-derived polyclonal IgA. In the first study, laboratory-adapted HIV_IIIB_ was tested in U937 cells in the presence of either IgA from seronegative individuals or from seropositive individuals at different stages of HIV disease. Only modest increases of virus replication by serum IgA were seen that could be blocked by preincubating the cells with an anti-FcαR mAb [80]. Similar, low-level enhancement of viral replication by total serum IgA from seropositive individuals was observed in primary monocytes with HIV_BaL_, a primary isolate. This effect could be suppressed by pretreating the cells with IgA isolated from HIV-seronegative individuals [81]. Together, these studies implied FcαR-mediated enhancement of HIV infection; however, the relevance of these in vitro findings to mucosal HIV transmission is uncertain.

### Humoral immune responses in the RV144 trial: Was protective IgG outcompeted by IgA?

The RV144 phase III trial had a modest 31.2% efficacy in preventing HIV acquisition [82]. Follow-up studies to determine correlates of risk of infection demonstrated that plasma IgG targeting the variable loops 1 and 2 (V1-V2) of HIV Env was associated with a decreased risk of HIV acquisition. In contrast, plasma anti-HIV Env IgA was linked to an increased risk of infection [83]. Another factor favoring beneficial outcomes was ADCC activity mediated by plasma IgG. More recently, Tomaras et al. [84] reported that antibodies recognizing epitopes within the first constant region (C1) of HIV gp120 mediated most of the ADCC activity observed. These authors went on to show that two different anti-C1 IgA2 mAbs interfered with the IgG1-mediated ADCC activity, therefore blocking a beneficial vaccine-induced antibody effector function. Follow-up clinical studies (RV305 and RV306) using the same immunogens as in RV144 have been initiated to analyze vaccine-induced, mucosal antibody responses [84].

## Conclusions

In vitro studies have identified several protective anti-HIV mechanisms of IgA, including direct HIV neutralization, inhibition of transcytosis, intracellular virus neutralization, and excretion of infectious virus from the basolateral side of the mucosal barrier. Anti-HIV IgA also has effector mechanisms, such as ADCC and complement activation via the alternative and lectin pathways. Together, these various IgA actions represent defense mechanisms that could benefit the host.

Mucosal IgA responses against different viruses protect the host in various model systems [53,85-87]. The role of mucosal anti-HIV IgA in humans is less clear, although data from HEPS implied that mucosally produced IgA may be responsible, at least partially, for preventing systemic infection. Direct evidence for IgA-mediated prevention of mucosal virus transmission was obtained by passive immunization in SHIV-challenged macaques, where mucosally administered monoclonal dIgA1 completely protected most animals, in contrast to the dIgA2 version with the same epitope specificity; better protection was linked to better virion capture and prevention of transcytosis [53]. Active immunization of RMs with virosomes displaying HIV gp41 likewise linked prevention of persistent viremia to the induction of mucosal IgA responses capable of blocking transcytosis in vitro [74].

Potentially problematic aspects of anti-HIV IgAs include IgA-dependent enhancement of HIV infection in cultured cell lines and primary monocytes [80,81], although this enhancement was modest and was overcome by higher concentrations of autologous IgG. The relevance of these in vitro IgA characteristics to mucosal HIV transmission is unclear currently. Recent data from the RV144 trial have also raised concerns that plasma anti-HIV Env IgA responses deprive the host of the benefits of IgG-mediated ADCC directed against shared targets on Env [84]. The RV144 analysis only involved plasma IgA since no mucosal samples were available [83,84].

In summary, while passive and active immunization studies in macaques gave proof-of-concept that SHIV acquisition can be prevented by mucosal IgA, the jury is still out whether anti-HIV IgA responses will overall benefit or harm the host. Future research will need to dissect the role of anti-HIV IgA in the systemic circulation as well as in different mucosal compartments and clearly define which forms of anti-HIV IgA are involved at such sites at the time of virus exposure. As our review of existing data has shown, not all IgAs are created equal, and it is possible that the various forms of IgA can differentially affect HIV transmission or spread.

## Abbreviations

aa: Amino acid
ADCC: Antibody-dependent cellular cytotoxicity
Cα: Constant region α
C1: Constant region 1
CL: Constant region of the light chain
dIgA: Dimeric immunoglobulin A
FcαRI: Fc receptor αRI
HIV: Human immunodeficiency virus
HEPS: HIV-exposed persistently seronegative persons
IgA: Immunoglobulin A
IgG: Immunoglobulin G
i.m.: Intramucsular
i.n.: Intranasal
i.r.: Intrarectally
J chain: Joining chain
mAbs: Monoclonal antibodies
nmAbs: Neutralizing monoclonal antibodies
pIgR: Polymeric immunoglobulin receptor
RMs: Rhesus monkeys
SC: Secretory component
SIgA: Secretory immunoglobulin A
SHIV: Simian-human immunodeficiency virus
V1: Variable loop 1
VH: Variable region of the heavy chain
VL: Variable region of the light chain
